# D for dominant: porcine circovirus 2d (PCV-2d) prevalence over other genotypes in wild boars and higher viral flows from domestic pigs in Italy

**DOI:** 10.3389/fmicb.2024.1412615

**Published:** 2024-06-17

**Authors:** Giulia Faustini, Francesca Poletto, Riccardo Baston, Claudia Maria Tucciarone, Matteo Legnardi, Mariangela Dal Maso, Viviana Genna, Laura Fiorentini, Alessandra Di Donato, Simona Perulli, Mattia Cecchinato, Michele Drigo, Giovanni Franzo

**Affiliations:** ^1^Department of Animal Medicine, Production and Health, University of Padova, Legnaro, Italy; ^2^AULSS 8 Berica, Dipartimento di Prevenzione, Servizi Veterinari, Vicenza, Italy; ^3^Azienda Ulss 9 Scaligera, Verona, Italy; ^4^Istituto Zooprofilattico Sperimentale Della Lombardia E Dell'Emilia Romagna (IZSLER), Forlì, Forlì-Cesena, Italy; ^5^Azienda Sanitaria Territoriale Di Ascoli Piceno, Ascoli Piceno, Italy

**Keywords:** PCV-2, wild boar, molecular epidemiology, spatial epidemiology, phylodynamic, phylogeny, Italy

## Abstract

**Introduction:**

Porcine circovirus 2 (PCV-2) is a key pathogen for the swine industry at a global level. Nine genotypes, differing in epidemiology and potentially virulence, emerged over time, with PCV-2a, -2b, and -2d being the most widespread and clinically relevant. Conversely, the distribution of minor genotypes appears geographically and temporally restricted, suggesting lower virulence and different epidemiological drivers. In 2022, PCV-2e, the most genetically and phenotypically divergent genotype, was identified in multiple rural farms in North-eastern Italy. Since rural pigs often have access to outdoor environment, the introduction from wild boars was investigated.

**Methods:**

Through a molecular and spatial approach, this study investigated the epidemiology and genetic diversity of PCV-2 in 122 wild boars across different provinces of North-eastern Italy.

**Results:**

Molecular analysis revealed a high PCV-2 frequency (81.1%, 99/122), and classified the majority of strains as PCV-2d (96.3%, 78/81), with sporadic occurrences of PCV-2a (1.2%, 1/81) and PCV-2b (2.5%, 2/81) genotypes. A viral flow directed primarily from domestic pigs to wild boars was estimated by phylogenetic and phylodynamic analyses.

**Discussion:**

These findings attested that the genotype replacement so far described only in the Italian domestic swine sector occurred also in wild boars. and suggested that the current heterogeneity of PCV-2d strains in Italian wild boars likely depends more on different introduction events from the domestic population rather than the presence of independent evolutionary pressures. While this might suggest PCV-2 circulation in wild boars having a marginal impact in the industrial sector, the sharing of PCV-2d strains across distinct wild populations, in absence of a consistent geographical pattern, suggests a complex interplay between domestic and wild pig populations, emphasizing the importance of improved biosecurity measures to mitigate the risk of pathogen transmission.

## 1 Introduction

Porcine circovirus type 2 (PCV-2) has been known in pig farming since the 1990s. First identified in pigs showing post-weaning multisystemic wasting syndrome (PMWS), it was later recognized as a global, endemic pathogen, infecting not only farmed pigs but also wild boars, raising questions about its true origin and increasing prevalence (Allan et al., 1998; Harding et al., 1998). Currently, PCV-2 has been associated with numerous clinical syndromes with multifactorial onset, collectively named porcine circovirus diseases (PCVD) (Segalés, 2012). However, major damages are caused by subclinical forms, leading to decreased performances and potentially impacting animal responsiveness to other challenges (Segalés, 2012; Alarcon et al., 2013). Because of the economic losses afflicting farmers, decades after its discovery, PCV-2 is still of major interest in the field, driving the efforts of researchers and pharmaceutical industries to develop effective control and prevention strategies.

PCV-2 is a small virus, characterized by a single-stranded circular DNA genome of approximately 1.7 kb in length, containing at least 6 coding regions (open reading frames, ORFs) (Lv et al., 2014; Li et al., 2018). ORF2 is of major interest, as it encodes the capsid protein (Cap), the dominant immunogenic antigen of PCV-2. Being the gene with the highest genetic variability, ORF2 serves also for genotype characterization (Nawagitgul et al., 2000; Franzo et al., 2016a). Over the years, the remarkable evolutionary rate of PCV-2 has led to the emergence of different variants, with nine genotypes currently recognized (from PCV-2a to −2 h). However, only PCV-2a, PCV-2b, and PCV-2d are globally widespread and clinically relevant, whereas minor genotypes have been identified inconsistently, both geographically and temporally (Franzo and Segalés, 2018; Wang et al., 2020).

Although the co-circulation of major genotypes has been observed for decades, there have been several changes in their prevalence over time. From 1996 to the early 2000s, PCV-2a was the predominant genotype clinically affecting pigs, but it was later replaced by PCV-2b (first genotype shift), which was associated with more severe clinical signs. Subsequently, PCV-2d emerged and supplanted PCV-2b (second genotype shift), becoming the most prevalent genotype in many countries worldwide, whereas PCV-2b and PCV-2a gradually declined in frequency (Xiao et al., 2015; Franzo et al., 2016a). The potential higher virulence of PCV-2b and PCV-2d, coupled with the widespread use of PCV-2a-based vaccines, is suspected to be driving these epidemiological waves, although the exact reasons remain to be clarified (Franzo et al., 2016b).

In Italy, despite its centrality in the European pig trade, the transition to genotype PCV-2d occurred slightly later than in other countries (Xiao et al., 2016; Franzo et al., 2016a), likely due to the peculiarity of Italian pig breeding for cured meat production, that leads to a slower turnover of the swine herd. Until 2016, genotypes PCV-2b and 2d were still present at a comparable frequency in domestic pigs and wild boars, although PCV-2d was gradually becoming dominant (Franzo et al., 2020). In 2022, an epidemiological survey conducted in northeastern Italy on intensively and rurally raised domestic pigs confirmed the genotype replacement, at least in the domestic population (Faustini et al., 2023). However, the unexpected identification of the minor genotype PCV-2e in multiple rural farms suggested a more widespread circulation than initially suspected (Franzo et al., 2022b). As outdoor access is common in rural farms, the introduction from wild boar seemed likely, also considering the frequent detection of PCV-2 in these populations, in Italy and elsewhere (Jacobsen et al., 2009; Petrini et al., 2009; Barbosa et al., 2016; Dei Giudici et al., 2019; Amoroso et al., 2021). The wild population plays a pivotal role also for the epidemiology of another swine circovirus (i.e., porcine circovirus type 3, PCV-3) (Franzo et al., 2023), and some minor PCV-2 genotypes have been detected previously in neglected populations (Franzo et al., 2015a; Faustini et al., 2023). Moreover, the recent demographic expansion of wild boars in Italy calls for renewed attention to this pig category (Massei et al., 2015). The present study aimed to update the knowledge of PCV-2 epidemiology in wild boars, characterizing the circulating strains and comparing them with the ones commonly detected in the commercial and rural farming system, evaluating the potential contacts, interplays, and flow directionality.

## 2 Materials and methods

### 2.1 Sampling

Lungs and lymph nodes of wild boars were collected in 2022 and 2023 during the routine culling campaign performed in Verona and Vicenza provinces, located in the Veneto region.

Official veterinarians supervised tissue sampling, while animal anamnestic data were provided by hunters by filling out a mandatory form. In particular, the following metadata were collected for each subject: hunter, municipality, hunting date, hunting method, sampling altitude and landscape, and wild boar age, sex, and biometric measurements.

To evaluate the impact of significant geographic separation on viral dispersal in wild populations, available archives samples (lungs and lymph nodes) collected by Istituto Zooprofilattico Sperimentale di Lombardia ed. Emilia Romagna (IZSLER, Forlì, Italy) in the provinces of Forlì-Cesena, Ravenna, Rimini (located approximatively 135–197 km from Verona and Vicenza provinces) were included in the study. All wild boar samples from IZSLER included in the study were obtained as part of routine diagnostic procedures.

All samples were stored at −80°C until DNA extraction.

### 2.2 PCV-2 qPCR screening

Wild boar lungs and lymph nodes were mechanically homogenized after adding 10 mL of 1X PBS (phosphate buffer saline) to each gram of tissue. DNA was extracted from 100 μL of homogenate, using the Viral DNA/RNA Kit (A&A Biotechnology, Gdansk, Poland), according to the manufacturer’s instructions. Extracted DNA was stored at −80°C until further processing.

The screening for PCV-2 presence was performed by real-time PCR (qPCR) using a DyNAmo Flash Probe qPCR kit (Thermo Fisher Scientific, Waltham, MA, United States) as described by Franzo et al. (2020).

### 2.3 PCV-2 sequencing and phylogenetic analysis

Amplification and sequencing of the ORF2 gene were attempted on all PCV-2 positive samples, using different primer pairs previously designed (Franzo et al., 2015b). All the PCRs were performed using Biometra TAdvanced^®^ Thermal Cycler (Analytik Jena GmbH, Jena, Germany) and Invitrogen^™^ Platinum^™^ II Taq Hot-Start DNA polymerase (Thermo Fisher Scientific, Waltham, MA, United States), according to the following protocol: 5 μL of extracted DNA was added to a standard reaction containing 1X Platinum^™^ II PCR buffer, 0.6 μM of each primer, 0.2 mM of each dNTP, and one unit of Platinum^™^ Taq Hot-Start DNA Polymerase. Sterile pure water was added up to a final volume of 25 μL. The thermal protocol was set according to the manual instructions of the kit. The amplification and specificity of bands were verified through SYBR™ Safe (Thermo Fisher Scientific, Waltham, MA, United States) stained agarose gel electrophoresis runs. All positive amplicons were purified using Applied Biosystems^®^ ExoSAP-IT PCR Product Cleanup Reagent (Thermo Fisher Scientific, Waltham, MA, United States). The same PCR primers were used for Sanger sequencing of the amplicons in both directions at Macrogen Europe (Milan, Italy).

Chromatograms were visually evaluated, appropriately trimmed using 4Peaks (Nucleobytes B.V., Aalsmer, The Netherlands), and then assembled to generate consensus sequences in ChromasPro (Technelysium Pty Ltd., South Brisbane, Australia). Obtained ORF2 sequences were aligned using the MUSCLE method implemented in MEGA X (Edgar, 2004; Kumar et al., 2018) and trimmed preserving the complete ORF2 region.

Recombination analysis was performed on complete ORF2 sequences using the Genetic Algorithm for Recombination Detection method (GARD) implemented in Datamonkey (Kosakovsky Pond et al., 2006; Weaver et al., 2018). A maximum-likelihood (ML) phylogenetic tree was inferred using MEGA X for genotyping purposes after alignment with the reference dataset suggested by Franzo and Segalés (2018), adopting the substitution model with the lowest Bayesian Information Criterion (BIC). To assess the robustness of the inferred clades, 1,000 bootstrap replicates were performed. To broaden the dataset, the same analysis was performed also including strains for which only partial ORF2 sequences were obtained.

Episodic diversifying selection was investigated using the Mixed Effects Model of Evolution (MEME) (Murrell et al., 2012), with a significance value set to *p-*valu*e* < 0.01.

### 2.4 Analysis of viral occurrence and genetic distance among strains at the province level

To calculate the odds ratio of infection among provinces, a logistic regression model was fitted using the PCV-2 qPCR frequencies as the dependent variable and the province as the independent one. The differences in viral titers of positive samples among provinces were assessed through ANOVA. Titers were log10 transformed to respect the normality and homoscedasticity assumptions. All statistical analyses were performed using R. The level of statistical significance for all considered tests was set at *p* < 0.05.

To compare the genetic diversity of PCV-2 strains circulating in the different provinces, five different sets of aligned complete sequences were created, one for each province (i.e., Forlì-Cesena, Ravenna, Rimini, Verona, and Vicenza), and related pairwise p-distances were calculated using MEGA X. To analyze the heterogeneity among PCV-2d strains, the same analysis was performed after excluding other genotypes from respective alignments. The difference among provinces in terms of mean pairwise p-distance was evaluated by the Kruskal–Wallis test. Dunn’s test with Bonferroni correction was performed as a *post-hoc* analysis for multiple comparisons.

To investigate the potential correlation between genetic diversity within a province and the corresponding density of wild boar populations, the Pearson correlation coefficient was calculated. The wild boar density in each province was obtained with the following approach in QGIS 3.22 Białowieża LTR: in the study area, a buffer of 10.38 km [average dispersal range of adult wild boars according to Keuling et al. (2010) from the borders of each municipality was calculated; the average density of wild boars within each obtained buffer was extracted from the wild boar density raster estimated by Pittiglio et al. (2018)]; the average density was used as an aggregated measure for each province.

All statistical analyses were performed using R. The level of statistical significance for all considered tests was set at *p*-value <0.05.

### 2.5 Spatial and phylogeographic analysis

#### 2.5.1 Cluster analysis of PCV-2 positive wild boars

To detect the presence of significant clusters of PCV-2-positive wild boars, a purely spatial analysis was implemented on SaTScan^®^ for each province (Kulldorff and Nagarwalla, 1995; Kulldorff, 1997). For each province, the results of the qPCR screening were aggregated at the municipal level and reported as binary data (positive = 1, negative = 0). The coordinates of the centroids of each municipality were extracted from the proper shapefiles^1^ using QGIS 3.22 Białowieża LTR, using a projected reference system (i.e., Datum ETRF90, EPSG:7917 reference system), as required by SaTScan^®^ software. Three separate datasets were prepared for each province, reporting the cases (PCV-2-positive municipalities), controls (PCV-2-negative municipalities), and centroid coordinates of the considered municipalities. A Bernoulli probability model was implemented, scanning for areas with high rates of cases (maximum spatial cluster size of 50% of the population at risk, and restricting clusters to at least 2 cases), and a maximum number of Monte Carlo replications of 9,999. The results obtained were then mapped using QGIS 3.22 Białowieża LTR. Similarly to what was performed at the province level, the presence of a significant difference in terms of mean pairwise p-distance between the SaTScan-detected cluster and the surrounding area was evaluated using the Kruskal–Wallis test.

#### 2.5.2 Analysis of the spatial structure of PCV-2d strain genetic diversity

A preliminary evaluation of the relationship between phylogeny and spatial distribution was obtained mapping the ML phylogenetic tree calculated using the sequences obtained from the sampled wild boars on the study area landscape using the *phytools* library in R.

To evaluate the spatial structure of PCV-2d strains continuously and assess the potential presence of spatial autocorrelation/dependence (i.e., strains spatially closer tend to be genetically more similar), a semi-variogram was built by plotting the semi-variance of p-distance between all possible strains spaced a constant distance apart, against geographic distance, using the *phylin* library in R. The genetic matrix was calculated using MEGA X, while the geographical distance matrix was generated using the *dist* function from R base functions after providing geographical coordinates for each strain (i.e., centroid coordinates of the municipality) on the WGS84 datum, EPSG:4326 reference system. A lag size set equal to 0.06 was considered appropriate for the creation of a manageable semi-variogram to facilitate interpretation. The Mantel test was performed to test the correlation between the two matrices.

#### 2.5.3 PCV-2d viral strains dispersal among Italian geographic areas and animal categories

All available PCV-2 ORF2 sequences from Italy were downloaded from GenBank, recording region, province, animal category (i.e., wild boar, rural reared pig, and intensively raised pig), and date/year of sampling when available. Once genotyped according to the classification proposed by Franzo and Segalés (2018), all the strains belonging to PCV-2d were collected in an independent dataset together with PCV-2d strains from wild boars analyzed in this study.

The migration among Italian regions and provinces was reconstructed by performing a discrete state phylogeographic analysis, as described by Lemey et al. (2009). More in detail, the selected datasets were analyzed to reconstruct several population parameters, including time to the most recent common ancestor (tMRCA), evolutionary rate, and viral population dynamics using the Bayesian serial coalescent approach implemented in BEAST 1.10 (Suchard et al., 2018). For each dataset, the nucleotide substitution model was selected based on the BIC score calculated using JmodelTest (Darriba et al., 2012). The molecular clock was selected to calculate the marginal likelihood estimation through path-sampling and stepping-stone methods, as suggested by Baele et al. (2012). The non-parametric Bayesian Skygrid was selected to reconstruct viral population changes over time (relative genetic diversity: Effective population size generation time; N_e_ τ) (Hill and Baele, 2019). A discrete state phylogeographic analysis was also performed as described by Lemey et al. (2009), implementing an asymmetric migration model with Bayesian stochastic search variable selection (BSSVS), allowing to identify the most parsimonious description of the spreading process and calculating a Bayesian factor (BF) indicative of the statistical significance of the inferred migration path between geographic areas. The log and tree files were merged using logcombiner after the removal of a burn-in of 20%. The results were analyzed using Tracer 1.7 and accepted only if the estimated sample size (ESS) was greater than 200, and the convergence and mixing were adequate. Parameter estimation was summarized in terms of mean and 95% highest posterior density (HPD). Maximum clade credibility (MCC) trees were constructed and annotated using TreeAnnotator (BEAST package). SpreaD4 (Bielejec et al., 2016) was used to calculate the BF associated with each migration route. All non-zero transition rates among countries were considered significant if the calculated BF was greater than 10.

The viral effective population size (Ne) and migration rates among animal categories (i.e., commercial, rural, and wild boar) were jointly estimated using the Bayesian structured coalescent analysis implemented in the package MultitypeTree of BEAST 2.7 (Vaughan et al., 2014; De Maio et al., 2015; Bouckaert et al., 2019). A 100 million generation Markov chain Monte Carlo (MCMC) run was performed on the dataset including all Italian ORF2 sequences originating from subjects whose category was known, sampling model parameters and trees every 10,000 generations. The nucleotide substitution model was selected based on the Bayesian Information Criterion (BIC), while a relaxed lognormal molecular clock was implemented. The results were processed as previously described.

To evaluate the difference between mainland Italy and Sardinia, an Italian island that featured a complex interplay between rurally raised breeds and wild boars, the same analysis was repeated excluding strains collected in this region.

## 3 Results

### 3.1 Sample location and PCV-2 screening results

**Figure 1:**
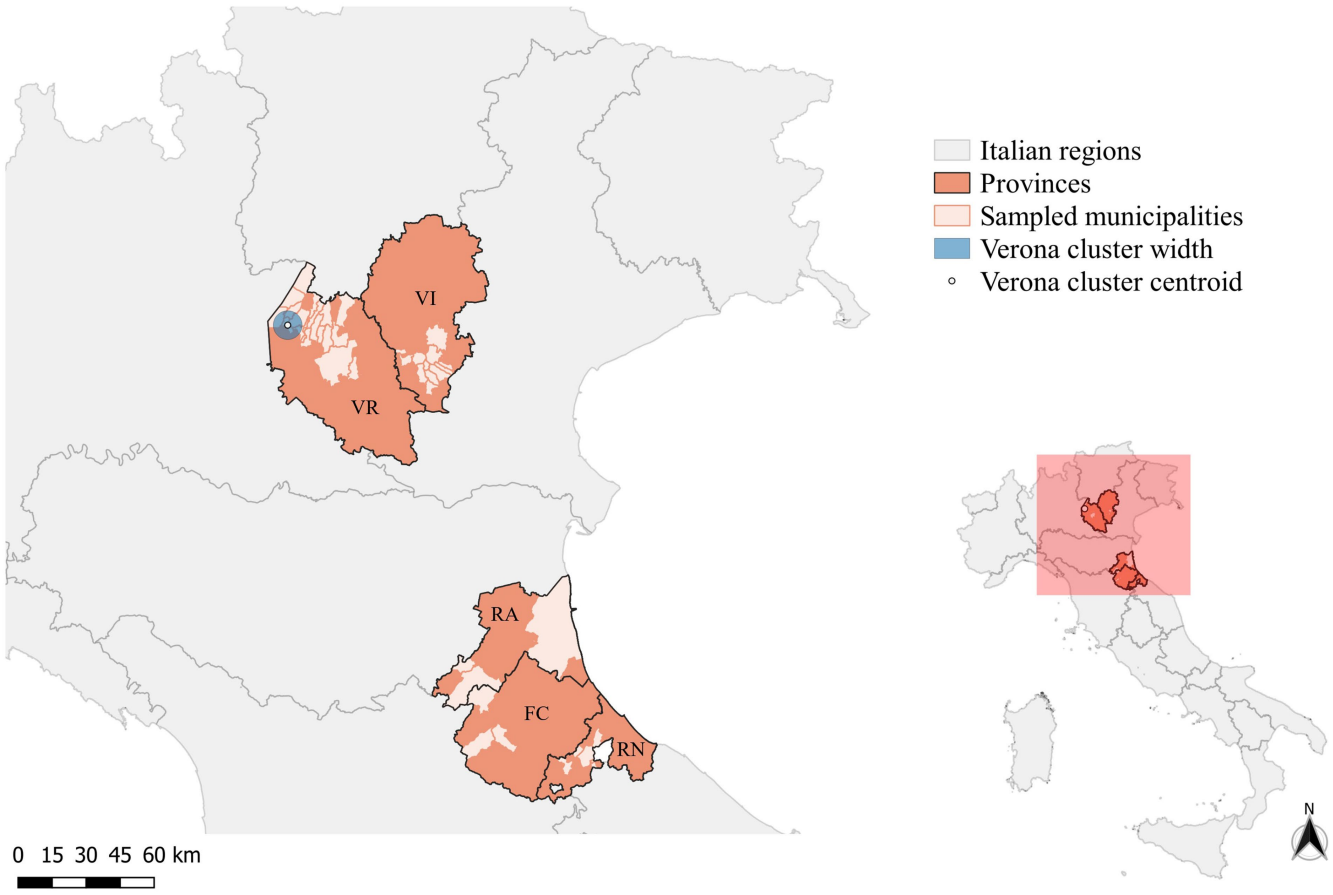
Map of the provinces of Forlì-Cesena (FC), Ravenna (RA), Rimini (RN), Verona (VR), and Vicenza (VI) showing the municipalities where wild boars included in the study were sampled. The circle indicates the significant cluster of PCV-2-positive wild boars (blue-filled circle).

Overall, 122 wild boars were included in the study; 112 were sampled during the licensed culling campaign conducted in 18 municipalities of Verona (*n* = 83) and 12 of Vicenza (*n* = 29) provinces. The municipality of two wild boars hunted in Verona was unknown. From the Emilia-Romagna region, the IZSLER provided 10 archived samples of wild boars collected in 2022 from three municipalities of Forlì-Cesena (*n* = 3) and Rimini (*n* = 3), and from four municipalities (*n* = 4) of Ravenna (Figure 1).

In total, 99 (81.1%, 99/122) samples tested positive for PCV-2 by qPCR. Of those, 66 were from Verona (79.5%, 66/83), 23 from Vicenza (79.3%, 23/29), 3 from Forlì-Cesena (100%, 3/3), 4 from Ravenna (100%, 4/4), and 3 from Rimini (100%, 3/3). No effect of the collection province on the likelihood of a positive qPCR result for PCV-2 was observed (*p*-value >0.05). Forlì-Cesena was the province with the greatest mean viral titer, followed in order by Rimini, Ravenna, Verona, and Vicenza. The mean titer reported in Vicenza was statistically lower than those in all other provinces (*p*-value <0.01), and the one reported in Forlì-Cesena was greater for both Verona and Vicenza (*p* < 0.01).

Considering the spatial distribution of PCV-2-positive wild boars, the cluster analysis performed on SaTScan^®^ identified only one significant cluster (*p*-value >0.05), including the municipalities of Costermano sul Garda, Torri del Benaco, Caprino Veronese, and Rivoli Veronese (Figure 1). In an area of 6.12 km radius, 23 cases were reported, against an expected case number of 18.51 (Relative risk (RR) =1.37). No significant clusters were identified, in the province of Vicenza. As all the sampled wild boars from Forlì-Cesena, Ravenna, and Rimini were positive for PCV-2, and no control dataset could be built, the cluster analysis was not performed for these provinces.

### 3.2 PCV-2 genotypes in wild boars

A total of 81 ORF2 sequences, 50 complete (acc. Numbers OR966756–OR966805) and 31 partial sequences (acc. Numbers OR966806–OR966836), were obtained (Supplementary material S1).

**Figure 2:**
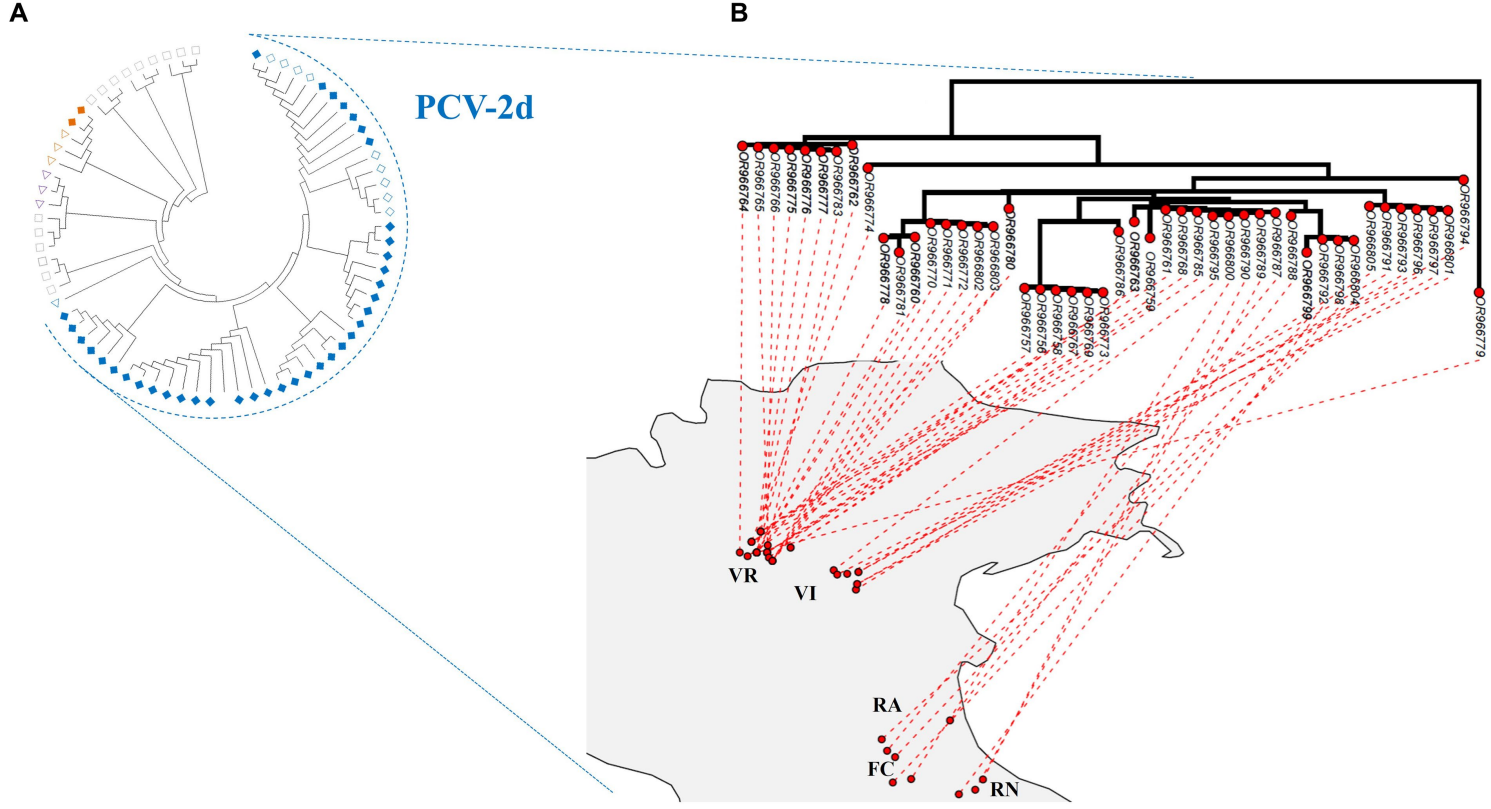
**(A)** Maximum-likelihood phylogenetic tree estimated on the complete ORF2 alignment of PCV-2 strains obtained in the present study, marked with a rhombus. Emilia-Romagna sequences are labeled with empty rhombus, whereas Veneto sequences are color-filled. Reference sequences whose genotypes have been detected in the present study have been marked with a triangle, whereas those not identified (−2c, −2e, −2 f, −2 g, and −2 h) with a rectangle. Genotypes have been color-coded: PCV-2a in purple, −2b in orange, and −2d in blue. Accession number, collection host, region, province, and date are reported in detail in Figure S1, in addition to bootstrap support. **(B)** Maximum-likelihood phylogenetic tree of PCV-2d circulating strains projected on analyzed northeastern Italy provinces: Forlì-Cesena (FC), Ravenna (RA), Rimini (RN), Verona (VR), and Vicenza (VI). Strains located in the geographical cluster detected by SaTScan v10.1.2. are reported in bold.

No recombination and episodic diversifying selection were detected, respectively, by GARD and MEME. According to the reference dataset proposed by Franzo and Segalés (2018), the estimated ML phylogenetic trees classified 78 strains as genotype PCV-2d, 2 as PCV-2b, and 1 as PCV-2a (Figure 2A; Supplementary material S2; partial sequences are included in Supplementary material S3). The PCV-2b strains were detected in wild boars hunted in Verona, whereas the unique PCV-2a strain was obtained from a wild boar sampled in Vicenza. The phylogenetic analysis showed an apparent geographical clustering by province (Figure 2B and Supplementary material S2).

### 3.3 Genetic diversity of circulating strains

The mean pairwise genetic distance among all obtained complete sequences was 1.07% [interval: 0.00–6.55%]. Considering only the PCV-2d genotype, overall, the mean p-distances was 0.63% [interval: 0.00–1.42%]. When PCV-2d strains pairwise p-distance was calculated independently for each province, Verona showed a greater genetic diversity: the mean pairwise genetic distance was 0.72% in Verona [interval: 0.00–1.42%], 0.13% in Vicenza [interval: 0.00–0.28%], 0.37% in Forlì-Cesena [interval: 0.00–0.56%], 0.28% in Ravenna [interval: 0.14–0.42%], and 0.28% in Rimini [interval: 0.00–0.43%]. The mean genetic distance was different among provinces (*p*-value <0.001); in particular, the mean genetic distance of PCV-2d strains from Verona was significantly greater than the one of PCV-2d strains of other considered provinces, except for PCV-2d strains from Forlì-Cesena.

The mean p-distance of PCV-2d strains circulating in the Verona municipalities belonging to the identified spatial cluster [mean: 0.58%, interval: 0.00–1.13%] was significantly different from the mean p-distance of the other PCV-2d strains circulating in the Verona municipalities (*p*-value <0.001), revealing a greater genetic similarity among the strains belonging to the spatial cluster.

However, as a non-significant correlation coefficient of *r* = −0.03 (*p*-value >0.05) was obtained, it was not possible to demonstrate if the difference in the genetic distance of strains circulating in different provinces correlated with a denser wild boar population in the corresponding area.

### 3.4 Spatial structure of PCV-2d strains genetic diversity

The phylogenetic tree mapped on the study area revealed the co-circulation of strains belonging to different clades in Verona, while in the other provinces, the majority of circulating strains belonged to one clade (Figure 2B).

**Figure 3:**
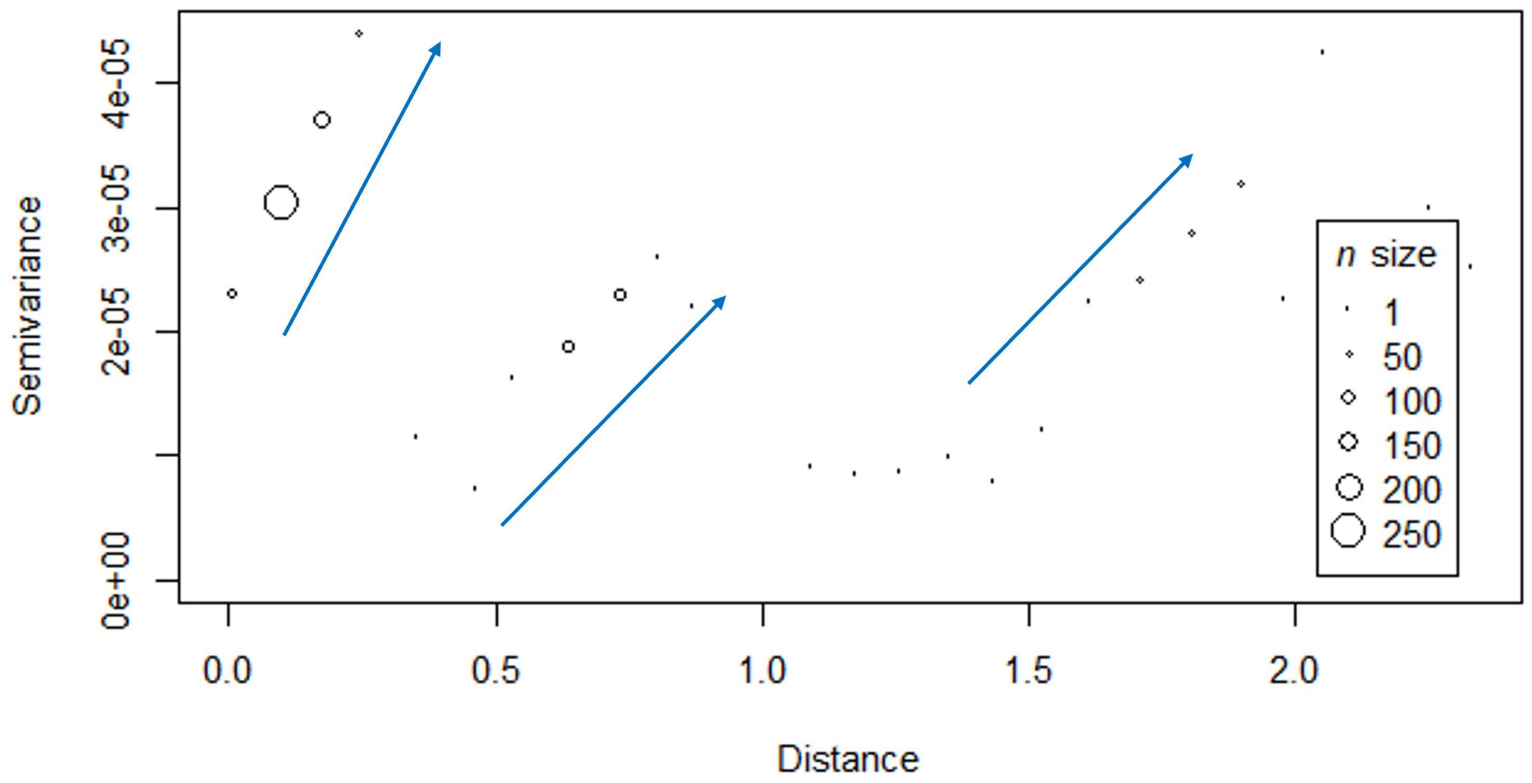
Semi-variogram displaying the relationship between geographic distance (in decimal degree, calculated on the WGS84 datum, using EPSG:4326 coordinate reference system) and genetic distance (semi-variance of genetic distance). The blue arrows highlight the major shifts in genetic distance occurring at three different spatial distance intervals: approximately between 0.0–0.3, 0.3–0.8, and 1.0–2.0 degrees (longitude and latitude on the WGS84 datum, EPSG:4326 reference system), which in kilometers correspond to 0–23.87, 23.87–70.38, and 109.65–192.23 km.

The semi-variogram obtained exhibited a distinct pattern characterized by three stepwise increases in the genetic distance between PCV-2d strains. Major shifts in genetic distance occurred at three different spatial distance intervals: approximately between 0.0–0.3, 0.3–0.8, and 1.0–2.0 degrees (distance calculated from coordinates in longitude and latitude, on the WGS84 datum, EPSG:4326 reference system), which in kilometers correspond to 0–23.87, 23.87–70.38, and 109.65–192.23 km (Figure 3). No statistically significant relationship between genetic distance and geographic distance was demonstrated (*r* = −0.03295, *p*-value >0.05).

### 3.5 Structured coalescent analysis with PCV-2d Italian sequences

The dataset used for the analysis performed in BEAST included a total of 234 PCV-2d strains collected in a time span from 2010 to 2023. More specifically, 186 sequences were downloaded from GenBank, whereas 48 sequences were obtained from this study. In terms of animal category, 83 strains originated from wild boars, 19 from domestic pigs reared in a rural setting (rural pigs), and 132 from intensively raised domestic pigs (industrial pigs). The majority of the sequences belonged to Veneto (*n* = 92), followed by Sardinia (*n* = 63), Lombardy (*n* = 54), Emilia-Romagna (*n* = 14), Piedmont (*n* = 5), Basilicata (*n* = 4), and Friuli-Venezia Giulia (*n* = 2).

The coalescent Bayesian analysis performed on Italian PCV-2d strains estimated a recent origin for this genotype in Italy, set to 2001.74 (95HPD:1993.95–2007.89). The analysis of viral population dynamics highlighted a progressive growth since its origin, with a slowdown around 2010–2015, followed by a more marked rise thereafter, although with some fluctuations (Supplementary material S4).

The phylogeographic analysis revealed the absence of a strong clustering both regionally (Supplementary material S5) and within category (Supplementary material S6), as strains collected in different regions/categories were interspersed in the phylogenetic tree, and several migration events were inferred over time. Sardinian strains showed a slightly higher clustering tendency, although also in this case exceptions were noted. Accordingly, several statistically supported migration rates between regions were inferred. Lombardy, connected with Veneto, Sardinia, Piedmont, and Friuli-Venezia Giulia, played a major role, followed by Veneto (connected with Lombardy, Emilia-Romagna, and Friuli-Venezia Giulia) and Sardinia (with significant viral migrations with Emilia-Romagna and Basilicata).

**Figure 4:**
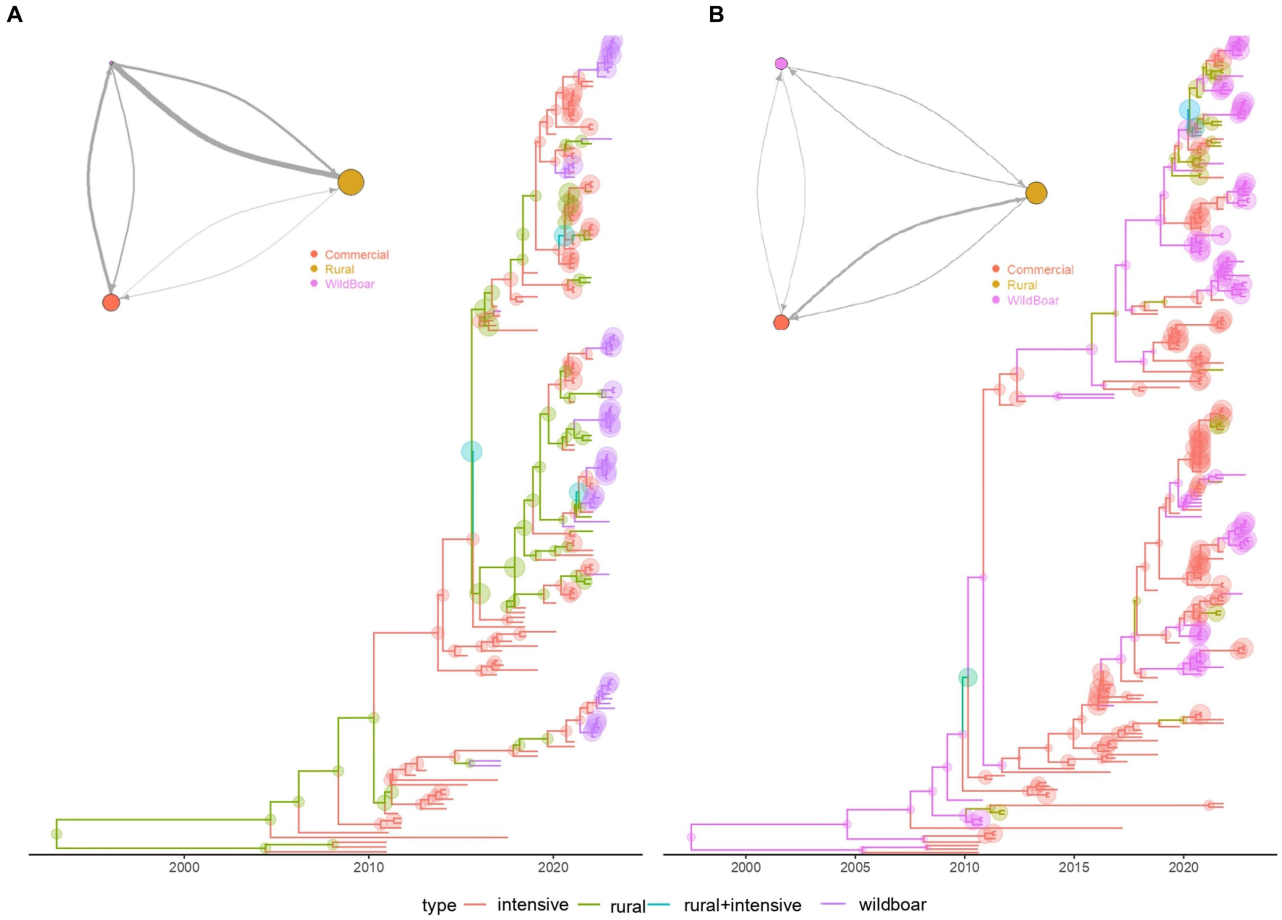
Structured coalescent-based phylogenetic tree of PCV-2d, **(A)** of mainland Italy only, **(B)** and including also Sardinia Island. Branch colors, as from the legend, mark the inferred animal category where the ancestral strain was circulating, while node size represents the posterior confidence of the inference. The network depicting the migration rate between animal categories is reported in the top left insert. Arrows and circle sizes are proportional to the inferred migration rate and population size, respectively.

The structured coalescent analysis performed on mainland Italy revealed a minor role of wild boars in the dynamic of PCV-2d, compared to rural and commercial pigs (Figure 4A). The estimated population size of PCV-2d in wild boars was approximately 6 and 4 times smaller than those circulating in rural and commercial pigs. Additionally, the main migration rates were from rural and commercial pigs toward wild boars, although the flux from wild to rural pigs was higher than the 1 from wild to commercial ones. Minimal flux seemed to occur between commercial and rural farms.

On the contrary, when Sardinia was included in the study the inferred PCV-2 size was similar among categories, as well as the intensity of migration rates (Figure 4B), suggesting a much stronger role and interconnection of these populations in Sardinia.

## 4 Discussion

The present study aimed to explore the potential presence of specific genotypes and strains peculiar to the wild context and to quantify the contribution of wild boars in the transmission fluxes of PCV-2, evaluating the contact network between different animal categories in Italy (Franzo et al., 2022b; Faustini et al., 2023); (Franzo et al., 2023).

The high PCV-2 frequencies described in this study confirmed the endemic nature of this pathogen in wild boars (Zhai et al., 2019; Franzo et al., 2020). Considering the temporal trend, a higher PCV-2 frequency (81.1%) was recorded for all the investigated provinces compared to what was previously described in other Italian territories, i.e., in Basilicata (27.0%), Campania (54.7%) and in other areas of Northern Italy (54.9%) (Franzo et al., 2020; Amoroso et al., 2021; Fanelli et al., 2022). Conversely, the overall PCV-2 frequency in this study was much closer to that described in Sardinia (82.6%) (Dei Giudici et al., 2019). In addition, the PCV-2d genotype shift was attested also in wild boars (Figure 2A; Supplementary material S2), mirroring what occurred in the Italian domestic sector (Faustini et al., 2023). This evidence suggested the higher fitness of this genotype, regardless of geographical location and animal category.

The increasing occurrence of PCV-2d in wild boars could reflect, at least partially, the spillover from the commercial sector. In fact, consistently with previous studies (Franzo et al., 2020), the structured coalescent analysis revealed that PCV-2d strains are transmitted mainly from domestic pigs to wild boars, describing a leaky pig farming sector, and the wild environment as a receiver of viral strains (Figure 4A). Therefore, PCV-2d prevalence in the wild population represented a direct and expected consequence, being widespread in domestic pigs for a long time.

The peculiar farming system in Sardinia had significant consequences on PCV-2 epidemiology (Supplementary material S5; Figure 4). On this island, domestic pigs are frequently raised in free-range facilities, easing frequent contacts and spillovers from and to wild boar (Mur et al., 2018). Accordingly, a more balanced flow with rural animals was identified, as well as a more prominent viral population in wild populations (Figure 4B). Despite limited contact between commercial and wild populations, an intermediator role of rural farms was estimated, as described for other swine pathogens (Figure 4B) (Franzo et al., 2023), contributing to the homogeneity of circulating viral strains in domestic and wild populations. As the movement of wild boars from the mainland can be excluded, novel strains in Sardinian wild boars have likely originated from domestic pigs, also imported from the mainland.

Interestingly, the directionality of the estimated PCV-2 viral fluxes was opposite to the one described for PCV-3, with the former mainly transmitted by domestic pigs, and the latter by wild boars (Figure 4A) (Franzo et al., 2020, 2023). The reasons behind these discrepancies might be biological, like a different host tropism, the virulence of circulating strains, the mutual competition for shared hosts, etc.

Across the provinces analyzed in this study, PCV-2 frequencies and genotype composition were comparable. Only Verona showed greater genetic heterogeneity among PCV-2d strains, attributable to the simultaneous circulation of two distinct clades (Figure 2B). Their presence in the Verona area could explain the stepwise pattern shown by the variogram, which described a greater genetic distance between more geographically distant strains in three separated spatial intervals: the observed intervals could be, respectively, related to the comparison between strains within Verona, between Verona and Vicenza, and between Verona and those circulating in Emilia Romagna provinces (Figure 3).

None of the two clades identified in Verona matched perfectly with the significant spatial cluster of PCV-2 positive wild boars identified in some Verona municipalities (Figure 2B). Therefore, the identified geographical cluster was most likely influenced by a data provision bias, ascribable to greater hunting pressure in those municipalities, or local features of wild population dynamics, rather than reflecting the circulation of a more infective strain in the spatial cluster area. Additionally, the most divergent clade belonged to a different evolutionary branch (Figure 2B; Supplementary material S6), indicating that the two clades in Verona originated from independent introduction events, rather than diverging for a different evolutionary boost of certain strains. Of note, also the wild boar strains within the PCV-2 clade circulating in all Veneto provinces were likely the result of multiple introduction events from domestic pig farms (Supplementary material S6).

Accordingly, considering the unlikely contacts between wild boar populations in the analyzed provinces/areas due to landscape morphology, and the minor role wild boars play in PCV-2 transmission in Italy (Figure 4A), the genetic similarity among strains from distinct wild populations is likely attributable to the domestic sector. Long-distance connections (e.g., trade) involving the domestic sector (Franzo et al., 2022a), followed by different introduction events in the wild, could justify the observed scenario.

This scenario confirms the ineffective compartmentalization in the Italian productive sector (Franzo et al., 2020, 2022a) and highlights the existence of interfaces between wild and domestic populations in different areas of Italy that strongly mandates an improvement in biosecurity strategies.

Commercial and rural pigs showed a wider variety of circulating strains than wild boars. The lower diversity of circulating strains in wild boars, despite their high positivity frequency, might originate from sporadic introductions of a limited number of strains mainly from neighboring farms. Subsequently, local strains may be maintained through interactions within the same wild boar population. If such strain selection reflects a different fitness in the wild population or it is merely due to chance would require further investigation (Franzo et al., 2016b).

According to phylogeographic results, Sardinia might have had several connections with mainland Italy, despite the trade restrictions due to African swine fever (at least until early 2023) (Mur et al., 2016). However, the relevance of Sardinia in the spread of PCV-2d in mainland Italy could have been biased by sequence availability. The inclusion of only Italian sequences in this study, as well as the obvious undersampling of circulating strains may have prevented the identification of intermediate steps, and it represents an unavoidable limitation of these studies (Franzo et al., 2015b). Moreover, data partial availability prevented the inclusion of all Italian regions in the present study. Nevertheless, the study provided a valuable depiction of Italian pig farming, from the most intensive and industrialized regions in Northern Italy to the less intensive systems present in the central and southern Italian regions, as well as the unique Sardinian system. Epidemiological updates from more regions and countries, involving rural farms and wild boars, are needed to provide a more comprehensive picture of current PCV-2 epidemiology in Italy and elsewhere.

The power of spatial analysis was likely reduced by the aggregation of the information by municipality, due to imprecise coordinates of hunted wild boars. Using precise spatial coordinates could enhance the understanding of spatial transmission and population dynamics and the role of other risk factors in the relationship between wild and domestic populations, which may vary spatially (Bengis et al., 2002; Massei et al., 2015).

The present study, along with others recently conducted, provides a comprehensive view of PCV-2 epidemiology in both domestic and wild pig populations in several Italian regions. Currently, Italian wild boars exhibit a high positivity frequency, confirming their susceptibility and potential for infection maintenance. Additionally, PCV-2d emerges as the predominant genotype, consistently with observations in commercial and rural pigs. The absence of strong geographical clusters, along with the high genetic similarity among PCV-2d strains in wild boars across distant geographical areas, and the results of the phylodynamic analysis support the frequency of multiple introductions from domestic pigs. Furthermore, this study marks the first attempt to approach PCV-2 epidemiology and diversity from a spatial perspective. Enhancing the spatial resolution by collecting more precise coordinates would allow us to investigate the domestic–wildlife interface and local geographic dynamics. Adopting a multidisciplinary approach integrating molecular and spatial methods would reveal patterns, networks, and drivers that might otherwise remain obscure.

## Data availability statement

The data presented in the study are deposited in the GenBank repository, accession numbers OR966756-OR966836.

## Ethics statement

Ethical approval was not required for the studies involving animals in accordance with the local legislation and institutional requirements because all samples from domestic pigs were obtained during routine clinical diagnostic activity at farm or slaughterhouse. Wild boar samples were achieved in the framework of planned culling campaign performed. No sampling or experimental procedure was specifically designed for the study. Written informed consent was not obtained from the owners for the participation of their animals in this study because all samples from domestic pigs were obtained during routine clinical diagnostic activity at farm or slaughterhouse. Wild boar samples were achieved in the framework of planned culling campaign performed.

## Author contributions

GFa: Conceptualization, Data curation, Formal analysis, Investigation, Methodology, Writing – original draft. FP: Data curation, Formal analysis, Writing – review & editing. RB: Methodology, Writing – review & editing. CT: Data curation, Visualization, Writing – review & editing. ML: Data curation, Formal analysis, Writing – review & editing. MDa: Resources, Writing – review & editing. VG: Investigation, Writing – review & editing. LF: Investigation, Resources, Writing – review & editing. AD: Investigation, Resources, Writing – review & editing. SP: Resources, Writing – review & editing. MC: Resources, Supervision, Writing – review & editing. MDr: Supervision, Writing – review & editing. GFr: Conceptualization, Data curation, Formal analysis, Funding acquisition, Investigation, Methodology, Project administration, Supervision, Writing – original draft.

## Footnotes

^1^ https://www.istat.it/
