# Supplementary material for: D for dominant: porcine circovirus 2d (PCV-2d) prevalence over other genotypes in wild boars and higher viral flows from domestic pigs in Italy
Source: Front Microbiol. 2024 Jun 17;15:1412615. doi: 10.3389/fmicb.2024.1412615 (PMC11215180; doi:10.3389/fmicb.2024.1412615)
Supplement: Supplementary file 1 [file Data_Sheet_1.pdf]

Supplementary Material

1 Supplementary Figures and Tables

1.1 Supplementary Figures

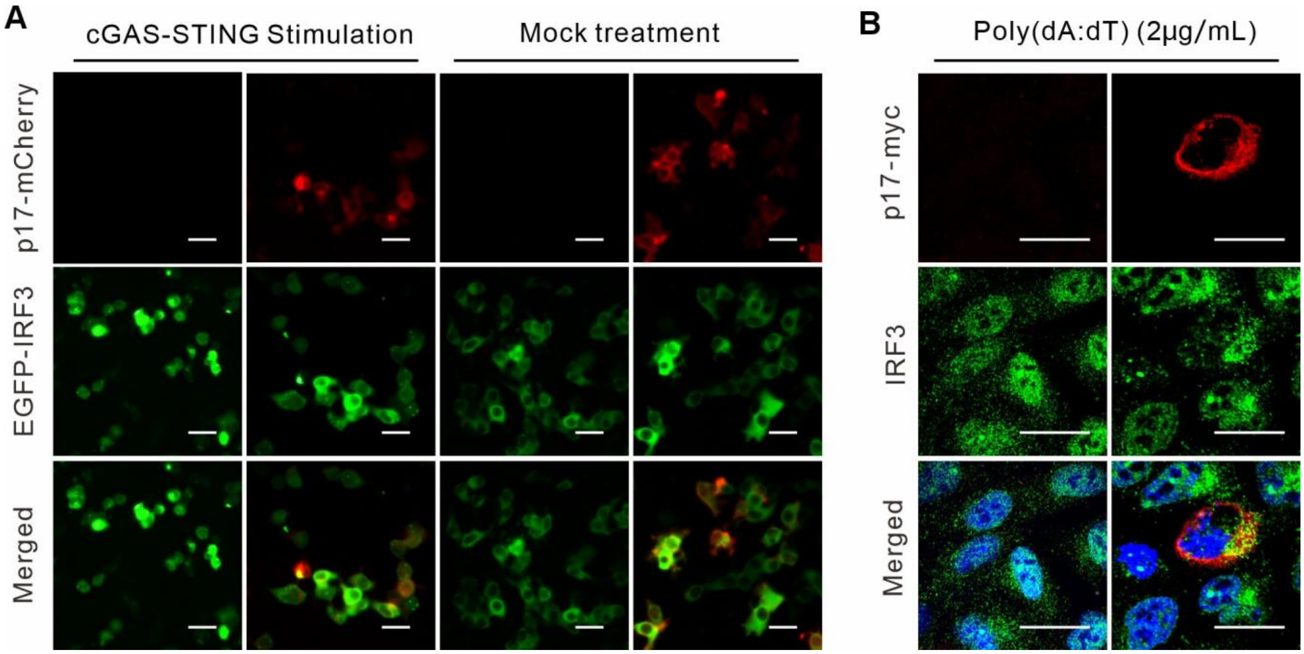

Supplementary Figure 1. ASFV p17 inhibited IRF3 nuclear translocation. (**A**) HEK293T cells were co-transfected with plasmids for HA-cGAS (0.4 μg), HA-STING (0.4 μg) or with empty vector pcDNA3.1 along with plasmids for EGFP-IRF3(0.2 μg) and p17-mCherry (0.1 μg). At 18 hours post transfection, the cells were visualized for localization change of IRF3 by an inverted fluorescence microscope. (**B**) HeLa cells were transfected to express p17-myc for 18 hours, and then stimulated with 2 μg poly(dA:dT) for another 6 hours. P17-myc were stained with rabbit anti-myc antibody and Alexa Fluor plus 488 anti-rabbit second antibody. IRF3 were stained with mouse anti-IRF3 antibody and Alexa Fluor plus 568 anti-mouse second antibody. Bar=10 μm.

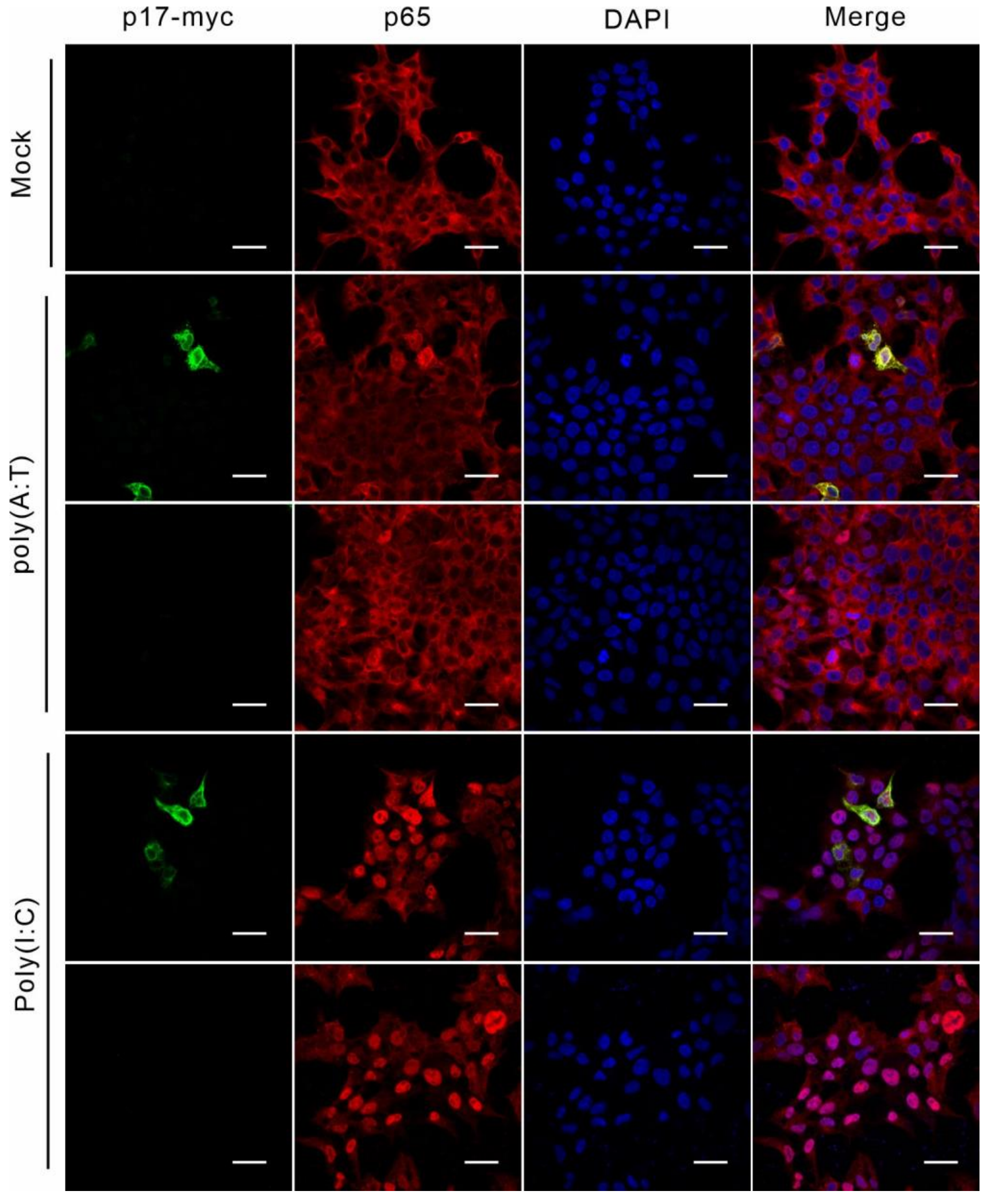

Supplementary Figure 2. HEK293T cells on coverslips in 12-well plates were transfected to express p17-myc and then stimulated with poly(dA:dT) (4 μg) or poly(I:C) (10 μg) for 6~12 hours. IFA was performed to stain indicated proteins with antibodies to p65 and myc epitope, followed by DAPI staining.

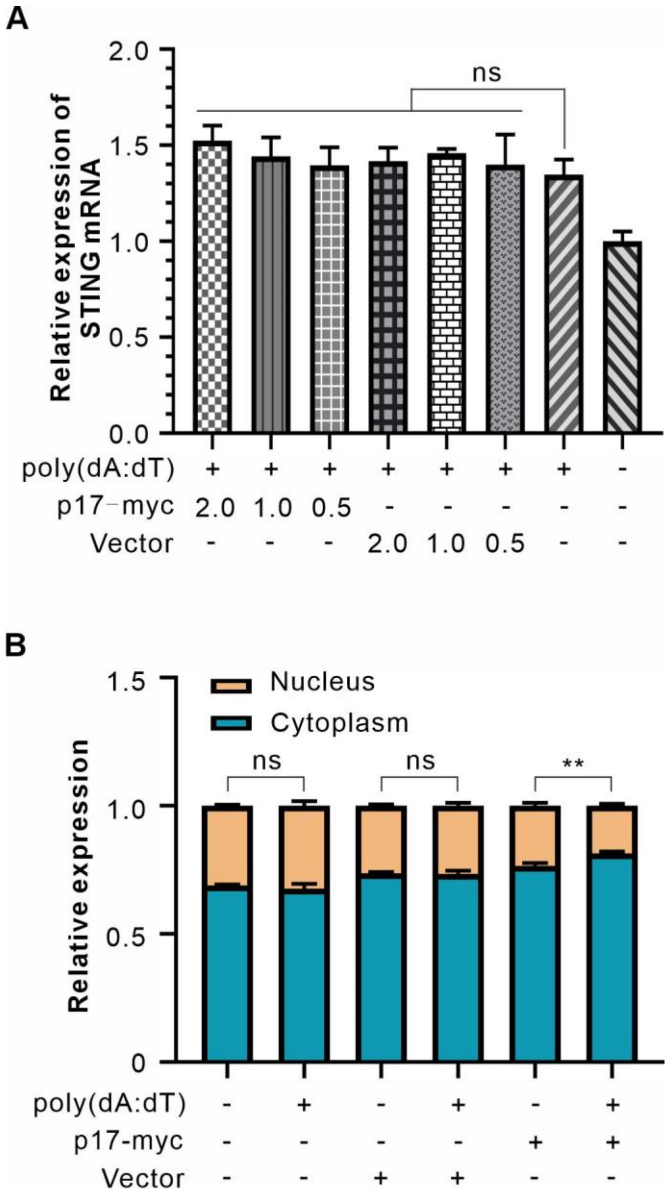

Supplementary Figure 3. (**A)** HeLa cells were transfected with p17-myc or an empty vector for 12 hours, then stimulated with poly(dA:dT) or mock-stimulated for another 12 hours. Total RNA was extracted, and STING mRNA was analyzed by qPCR and normalized against GAPDH. (**B**) Both cytoplasmic and nuclear RNA were extracted using the cytoplasmic and nuclear RNA purification kit (NORGEN) from HeLa cells treated as described above. The STING mRNA was detected using qPCR, and the relative expression was calculated with GAPDH and U6 mRNA as house-keeping genes, respectively.

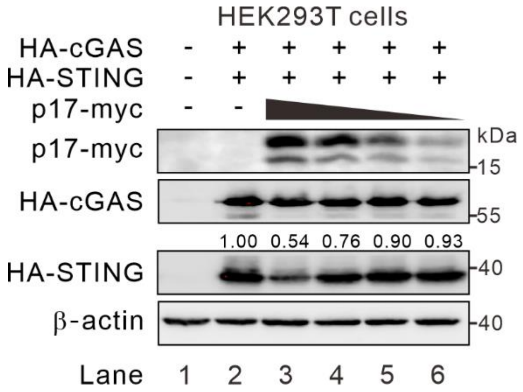

Supplementary Figure 4. Dose-dependent effect of p17 expression on degradation of exogenously STING in transfected HEK293T cells. HEK293T cells were transfected with HA-cGAS (0.3 μg) and HA-STING (0.3 μg), along with varying doses of p17-myc (0, 0.08, 0.15, 0.30 and 0.60 μg). At 24 hours post transfection, the cells were lysed and subject to Western blot analysis with antibodies to the indicated proteins.

1.2 Supplementary Tables

Supplemental Table 1. Primers and siRNA sequences used in this article

Primers	Sequence (5’-3’)
Pig-IFNβ-F	CATCCTCCAAATCGCTCTCC
Pig-IFNβ-R	CTGACATGCCAAATTGCTGC
Pig-ATCB-F	CAAGGACCTCTACGCCAACAC
Pig-ATCB-R	TGGAGGCGCGATGATCTT
Hu-IFNβ-F:	ATGACCAACAAGTGTCTCCTCC
Hu-IFNβ-R:	GGAATCCAAGCAAGTTGTAGCTC
Hu-STING-F:	GAGCAGGCCAAACTCTTCTG
Hu-STING-R:	CTGCTGTCATCTGCAGGTTC
Hu-GAPDH-F:	GAAGGGCTCATGACCACAGT
Hu-GAPDH-R:	GGATGCAGGGATGATGTTCT
Hu-U6-F:	CTCGCTTCGGCAGCACA
Hu-U6-R:	AACGCTTCACGAATTTGCGT
Pig-STING-F:	CTACCAGGAACCCACAGAGG
Pig-STING-R:	ATCTGAGCGGAGTGGAAGAG
Hu-cGAS-F:	TTCCAGATTACGCTGAATTCATGCAGCCTTGGCACGGAAAGG
Hu-cGAS-F:	TTCCAGATTACGCTGAATTCATGCAGCCTTGGCACGGAAAGG
Hu-cGAS-R:	GATCTGCTAGCTCGAGCTAAAATTCATCAAAAACTGGAAACTC
Hu-STING-F:	TTCCAGATTACGCTGAATTCATGCCCCACTCCAGCCTGCATCC
Hu-STING-R:	GATCTGCTAGCTCGAGCTAAGAGAAATCCGTGCGGAGAGGG
Hu-IRF3-F:	TTCCAGATTACGCTGAATTCATGGGAACCCCAAAGCCACGGAT
Hu-IRF3-R:	GATCTGCTAGCTCGAGCTAGCTCTCCCCAGGGCCCTGGAAAT
Hu-TBK1-F:	TTCCAGATTACGCTGAATTCATGCAGAGCACTTCTAATCATC
Hu-TBK1-R:	GATCTGCTAGCTCGAGCTAAAGACAGTCAACGTTGCGAAGG
Pig-cGAS-F:	TTCCAGATTACGCTGAATTCATGGCGGCCCGGCGGGGAAAGTC
Pig-cGAS-R:	GATCTGCTAGCTCGAGCTACCAAAAAACTGGAAATCCATTGTT
Pig-STING-F:	GGATGACGATGACAAGCTTCCCTACTCCAGCCTGCATCCATCC
Pig-STING-R:	GGGATGCCACCCGGGATCCCTAGAAGATATCTGAGCGGAGTGG
Pig-IRF3-F:	TTCCAGATTACGCTGAATTCATGGGAACTCAGAAGCCTCGGATC
Pig-IRF3-R:	GATCTGCTAGCTCGAGCTAGAAATCCATGTCCTCCACCAGGTCC

IRF3/5D-F:	TTCCAGATTACGCTGAATTCATGGGAACCCC
IRF3/5D-R:	GATCTGCTAGCTCGAGCTAGCTCTCCCCAGGGCCCTGGAAATCCATG CCCTCCACCAAGTCCTGCAGGTAGGCCTTGTACTGGTCGTCGTCGAG GTCGAGTGGGTGGTCGTTGTCAATGTGCAGGTCCAC
EGFP-IRF3-F:	AAGTCCGGACTCAGATCTCGAGGAACCCCAAAGCCACGGATC
EGFP-IRF3-R:	GTTATCTAGATCCGGTGGATCCCTAGCTCTCCCCAGGGCCCTGG
P17-mCherry-F:	GCTCAAGCTTCGAATTCGCCACCATGGACACCGAGACCAGCCCCCT
Pig-PR65A siRNA:	GCAACGAGGAUGUUCAGCUTT
Hu-PR65A siRNA:	UGGACAACGUCAAGAGUGATT

Supplemental Table 2. Interacting proteins with p17-myc identified by IP-MS (Top 20)

No.	Full name of interacted proteins with p17-myc	Abbreviation
1	Replication initiator 1	REPIN1
2	Eukaryotic translation initiation factor 3 subunit D	EIF3D
3	Phenylalanine--tRNA ligase beta subunit	FARSB
4	YTH domain containing 2	YTHDC2
5	Galactokinase 1	GALK1
6	Mitochondrial import inner membrane translocase subunit TIM50	-
7	Zinc finger C2HC-type containing 1A	ZC2HC1A
8	calcium/calmodulin-dependent protein kinase	CAMK2G
9	Thyroid transcription factor 1-associated protein 26	-
10	Filamin C	FLNC
11	Protein arginine N-methyltransferase 5	PRMT5
12	Hydroxyacyl-CoA dehydrogenase trifunctional multienzyme complex subunit beta	HADHB
13	Dimethylaniline monooxygenase [N-oxide-forming] 3	FMO3
14	G patch domain-containing protein 4 isoform 2	GPATCH4
15	Coatomer subunit delta	ARCN1
16	Exosome complex component RRP45	EXOSC9
17	Serine/threonine-protein phosphatase 2A 65 kDa regulatory subunit A alpha isoform	PPP2R1A (PR65A)
18	RNA-binding protein 4B	RBM4B
19	Chloride intracellular channel protein	CLIC1
20	WD repeat-containing protein 55	WDR55
